# Characterization of GMAW (Gas Metal Arc Welding) Penetration Using Ultrasonics

**DOI:** 10.3390/ma13102307

**Published:** 2020-05-17

**Authors:** Lu Zhang, Gorkem Okudan, Alexandra-Del-Carmen Basantes-Defaz, Ryan M. Gneiting, Sankaran Subramaniam, Didem Ozevin, Ernesto Indacochea

**Affiliations:** 1 Civil and Materials Engineering Department, University of Illinois at Chicago, Chicago, IL 60607, USA; zhang899@uic.edu (L.Z.); gokuda2@uic.edu (G.O.); Alexandria.basantes@gmail.com (A.-D.-C.B.-D.); jeindaco@uic.edu (E.I.); 2 Industrial Robotics, Deere and Company, Moline, IL 61265, USA; GneitingRyanM@johndeere.com (R.M.G.); SubramaniamSankaran@johndeere.com (S.S.)

**Keywords:** weld morphology, GMAW, linear ultrasonics, nonlinear ultrasonics

## Abstract

Welding defects such as lack of penetration, undercutting, crater crack, burn-through and porosity can occur during manufacturing. Assessing weld quality using nondestructive evaluation methods is important for the quality assurance of welded parts. In this paper, the measurement of weld penetration, which is directly related to weld integrity, is investigated by means of ultrasonics. Both linear and nonlinear ultrasonic methods are studied to assess their sensitivities to weld penetration. Welded plates with different penetration depths controlled by changing weld heat input are manufactured using gas metal arc welding (GMAW). Microscopic properties are assessed after the ultrasonic measurements are completed. Numerical models are built using the weld profile obtained from macrographs to explain the relationship between linear ultrasonic and weld penetration. A quantitative correlation between weld morphology (shape, width and depth) and the energy of linear ultrasonic signal is determined, where the increase of weld bead penetration exceeding the plate thickness results in decrease of the energy of the ultrasonic signal. Minimum detectable weld morphology using linear ultrasonics is defined depending on the selected frequency. Microhardness measurement is conducted to explain the sensitivity of nonlinear ultrasonics to both weld penetration and heterogeneity in weld. The numerical and experimental results show that the weld geometry influences the ultrasonic measurement other than the materials’ properties.

## 1. Introduction

Gas metal arc welding (GMAW) is the most commonly used industrial welding process since the 1940s in heavy industry manufacturing. In a GMAW process an electric arc is established between a consumable solid wire electrode and base metal using an externally supplied shielding gas. The intense heat from the electric arc melts a localized portion of the base metal and the consumable wire, which form a weld bead that is part of the weld joint. The shielding gas that is fed through a nozzle is used to establish the arc and for preventing the atmospheric contamination of the weld pool. Weld related parameters involved in the process which control weld quality, include type of shielding gas, gas flow rate, wire feed speed that depends on the applied current, voltage and travel speed. In GMAW, as with other welding processes, weld bead penetration is dependent on weld heat input, which depends on the type and thickness of metal alloy welded [1]. Assessing weld defects during manufacturing is vital to produce good quality welds, minimize defective products late in the manufacturing process and thus improve productivity. The assessment methods can be categorized as in-situ monitoring and post-production inspection. In-situ monitoring requires instrumenting the welding process to assess the quality during manufacturing, such as transient thermal and image analyses using optical data. Post-production inspection can be considered as destructive or non-destructive testing. The destructive methods include metallographic analyses, impact toughness tests, tensile testing or bending testing. Though those methods can measure the mechanical properties of a welded part, they require significant laboratory work to prepare and test the weld coupons. Nondestructive testing (NDT) methods applied to weld inspection include radiography, eddy current, magnetic particle testing and ultrasonics [2,3,4,5,6].

Ultrasonic testing (UT) has been widely applied to weld inspection due to its sensitivity to weld defects, its ability to inspect a large volume through ultrasonic scanner and its safe operation. Some examples of UT applications to GMAW reported in literature are listed in Table 1 [7,8,9,10,11,12,13,14,15,16,17,18]. The application, experimental configuration, material and weld types are summarized in this table. It shows that UT testing is versatile based on different testing objectives. Linear UT (LUT) is based on measuring the reflections from weld defects or scatters from heterogeneous medium that may be caused by different solidification morphologies or weld metal microstructures. Its resolution is limited to a half wavelength. Nonlinear UT (NLUT), on the other hand, can detect micro defects in materials in sub-wavelength. Most UT studies for weld characterization, as summarized in Table 1, are based on the experimental data. Therefore, building a physics-based correlation between UT and weld quality is needed. In addition, the authors applied both NLUT and LUT to characterize the weld bead morphology in gas tungsten arc weld (GTAW) [7]. The results show potentials of using UT to identify the weld penetration.

The objective of this study is to develop a quantitative nondestructive assessment of weld morphology for GMAW using ultrasonics. The authors developed a methodology to detect the weld quality of weld manufactured by gas tungsten arc welding (GTAW) [7]. The methodology is extended to GMAW in this study, which results in a more complex weld bead than GTAW. In order to realize an accurate evaluation of weld penetration, a quantitative relationship between the weld bead morphology and UT measurement is investigated. The sensitivity of geometric variability in weld bead is investigated experimentally and numerically. To build a realistic numerical model, an accurate weld shape is digitized using metallographic images and imported into the numerical models. The research provides a model-driven investigation of UT for assessing weld quality in addition to conventional experimental methods. The outline of this paper is as follows: the measurement method and the sample preparation are described in Section 2. Samples with different bead dimensions are manufactured using GMAW by adjusting weld heat input. The metallographic characterization, ultrasonic measurement and numerical results are presented in Section 3. After the UT measurement is completed, the samples are sectioned for metallurgical assessment of weld dimensions, which are digitized to input into numerical models. The microhardness analysis in Section 4 provides the evidence that material heterogeneity in weld bead is not the main reason that leads to the low-frequency UT detection. Section 5 summarizes the conclusions of this study.

## 2. Methods and Measurement Systems

### 2.1. Methods

**Figure 1:**
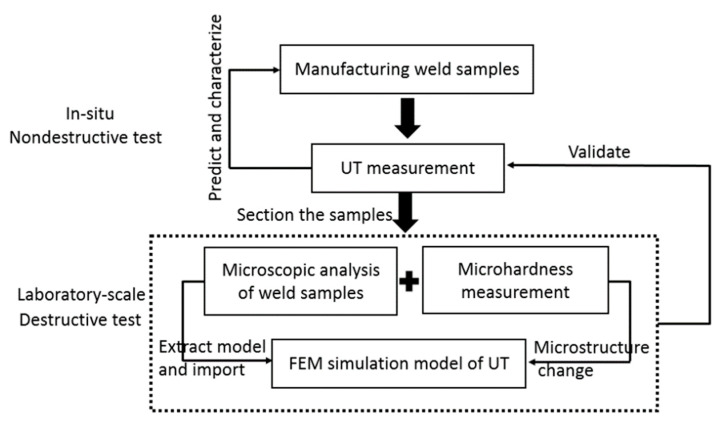
The connection of experimental results (UT and metallography) and numerical models (UT: Ultrasonic Testing, FEM: Finite Element Method).

The sequence of experimental and modeling methods included sample preparation with different weld bead dimensions, ultrasonic measurement, microscopic analyses and micro-hardness measurement at the weld sections, and then numerical models were built using the cross-sectional images obtained from macrographs. The link between each method is demonstrated in Figure 1. The numerical results assisted the interpretation of experimental results to build the correlation between the influence of weld shape to the UT output. By validating numerical results with experimental findings, an extensive model size could be built without experiments to obtain a reliable database for a variety of weld bead shapes.

### 2.2. Materials Preparation

**Figure 2:**
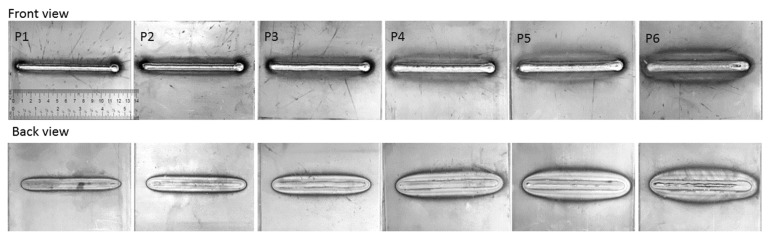
The front and back views of six samples.

Six weld samples were manufactured using robotic GMAW. The data set provided six data points to validate numerical models and build the correlation between UT and weld morphology. In order to reduce the uncertainties and obtain a better understanding of the weld bead morphology, bead-on-plate (BOP) welds were fabricated. The weld input parameters could be assumed to be directly related to the weld bead in BOP weld; and also, only one material was involved, which would also avoid the mismatch between different plates. The welding parameters including travel speed, current, voltage and gas flow rate were recorded using ARCAgent 3000P system and Centerpoint software (V9.01, Miller Electric Mfg. LLC, Appleton, WI, USA). The base material was an HSLA 350 (high strength and low alloy steel) of wt.% composition 0.08 C, 1.2 Mn, 0.3 Si, 0.05 Nb, 0.08 V. The coupon dimensions were 15 × 15 × 0.32 cm^3^. The filler material was a Lincoln ER70S6 wire (wt.% composition 0.06 C, 1.4 Mn, 0.8 Mn, 0.15 Ni, 0.15 Cr, 0.15 Mo, 0.03 V) and 0.89 mm (0.035 inch) in diameter. Each specimen was clamped onto the welding table to prevent bending or distortion of the plate, which negatively influences the UT measurement. The weld penetration was controlled by increasing the weld heat input gradually up to the point where burn-through occurs. The burn-through was monitored by examining the back of the plate. Table 2 summarizes the experimental variables. The front and back views of samples are shown in Figure 2. The width of weld line increased with the increase of weld heat input, and onset of burn-through was observed in P5. Further microscopic analyses were conducted to measure width, depth and area of weld bead.

### 2.3. Ultrasonic Measurement

**Figure 3:**
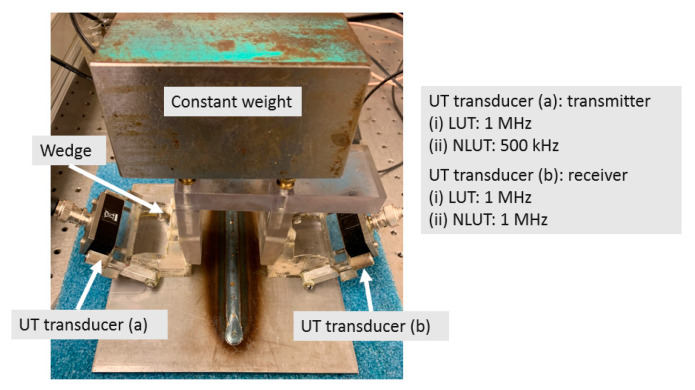
Experimental setups of LUT (Linear Ultrasonic Testing) and NLUT (Nonlinear Ultrasonic Testing).

The ultrasonic measurement consisted of linear and nonlinear ultrasonic testing (LUT and NLUT). The experimental setup for both methods was similar: Two normal beam transducers manufactured by Olympus-IMS Corp, Waltham, MA, USA were used in through-transmission mode; in which, two 1 MHz transducers were used as transmitter and receiver for LUT, and a 500 kHz transmitter and 1 MHz receiver were used for NLUT. The wavelength of 1 MHz frequency was approximately 1.5 mm, which was twice the thickness of base plate. In order to introduce a refracted shear wave into the weld plate, The plexiglass ultrasonic wedge was set at 57° for both LUT and NLUT. Two transducers were placed perpendicularly across the weld line with the constant space of 102.5 mm, shown in Figure 3. Light lubrication oil was used as couplant. In order to reduce the coupling error, a constant weight was applied. Moreover, each measurement was repeated six times with recoupling the transducers to study repeatability and find the measurement error due to coupling. A portable dual-channel tablet UT manufactured by Mistras Group Inc. (Princeton Junction, NJ, USA,) was used as data acquisition system. A 10-cycle and 16-cycle tone burst signal with 200-volt amplitude was generated as the excitation signal for LUT and NLUT, respectively. The data acquisition variables were sampling frequency as 100 MHz, digital filter as 200–2000 kHz and the average of 200 waveforms to improve the signal-to-noise ratio.

### 2.4. Metallographic Assessment

Once the UT measurement was completed, the samples were sectioned for metallographic assessment [20]. Metallographic assessments included visual observation of the macrostructure of the weld cross section and Vickers micro-hardness measurements. The cross section of the weld bead was examined using a stereomicroscope manufactured by Leica Microsystems (Leica Microsystems, Wetzlar, Germany). In order to visualize the weld bead, 8× amplification was selected. Additionally, the microstructures of each weld sample were examined using light metallograph. A Leica M400 micro-hardness tester (Leica Microsystems, Wetzlar, Germany) with Vickers indenters was used to assess the changes in hardness across the weldment (i.e., weld metal and heat-affected zone (HAZ)).

### 2.5. Numerical Models

**Figure 4:**
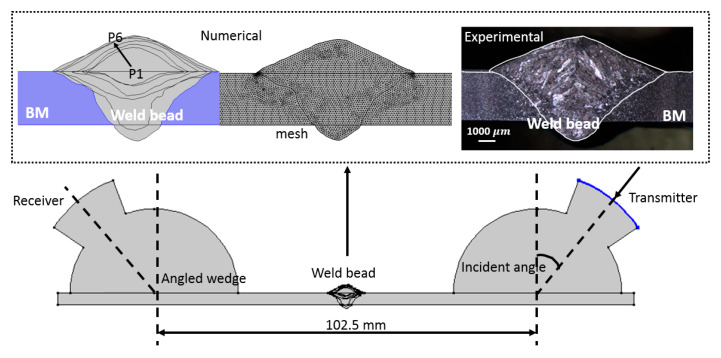
The schematic of the numerical model, (BM: Base Metal).

The numerical models were performed using COMSOL Multiphysics software (Version 4.2a, COMSOL Inc., Stockholm, Sweden). The purpose of the numerical models was to validate the experimental correlation between weld morphology and UT feature, such that additional models could be built to represent a variety of weld morphologies. This significantly reduced the experimental effort in the construction of the correlation curve. Most of the experimental details were considered in the models including loading, geometry and plexiglass wedge, as shown in Figure 4. For the accurate representation of weld shape in the numerical model, the geometry of weld bead was extracted from the stereomicroscope images by the image digitalizing software GetData Graph Digitize, and imported into the model. The excitation signal was considered as a line load (the same length as transducer width), and the average displacement at the receiving line was extracted. To reduce the computational time, the 2D plane strain model was adapted. The materials’ properties used in the numerical models are shown in Table 3. The transient analysis was conducted to simulate the ultrasonic wave propagation. Minimum mesh size of 0.15 mm and time step of 0.05 μs corresponded to 2 MHz frequency resolution. The loading function was the same as the excitation signal, which was the 10-cycle tone burst signal.

## 3. Results

### 3.1. The Correlation of Heat Input and Weld Morphology

The weld parameters (i.e., current, voltage and travel speed) were set to produce different heat inputs so as to assess weld penetration, weld bead width and, ultimately, burn-through. Any change in the prescribed parameters will affect the final weld quality and morphology. Weld bead morphology and heat-affected zone (HAZ) properties affected weld joint strength.

**Figure 5:**
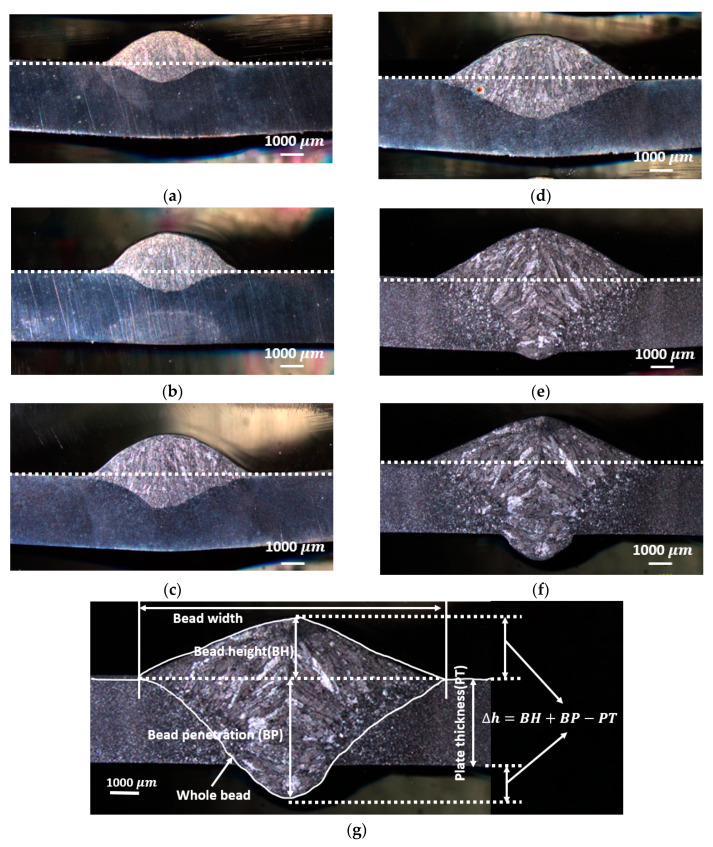
The macrophotographs of welds (**a**) P1, (**b**) P2, (**c**) P3, (**d**) P4, (**e**) P5, (**f**) P6 and (**g**) description of bead profile.

**Figure 6:**
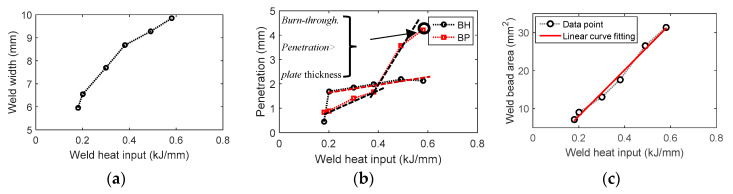
The relationship between weld heat input and (**a**) weld width, (**b**) penetration and (**c**) area of weld bead (BH: Bead Height, BP: Bead Penetration).

Figure 5 shows cross-sectional views of six samples obtained using stereomicroscope. The cross section of each weld sample was assessed by considering weld bead height (BH), weld bead width, bead penetration (BP) and thickness change (Δh), as illustrated in Figure 5g. Herein, Δh represents the maximum height change at weld bead, and its calculation is shown in Figure 5g. The detailed weld dimensions are summarized in Table 4. As it is observed in Figure 6, the increase of heat input resulted in the increase in weld penetration and weld width; however, the rates of increase were different. A linear relationship was observed between weld area and heat input, shown in Figure 6, which is expected since the increase in heat input results in larger amounts of weld wire and base metal being molten. A linear correlation between weld area and weld heat input was observed with the correlation coefficient R2 as 0.9897. There is also a well-defined linear correlation between weld heat input and weld bead penetration; however, the slope of the linear relationship between heat input and weld bead height is smaller, as seen in Figure 6b. With the increase of weld heat input, some burn-through was observed at the bottom surface, although no through-thickness hole occurred. The onset of burn-through started in P5, see in Figure 5e. Weld width, height and penetration were extracted from macrophotographs using a software called GetData Graph Digitize, which discretized the image. The discretized data points were transformed into coordinate information, which was imported into the COMSOL Multiphysics software to generate an accurate geometric model.

### 3.2. The Correlation between UT and Weld Morphology

**Figure 7:**
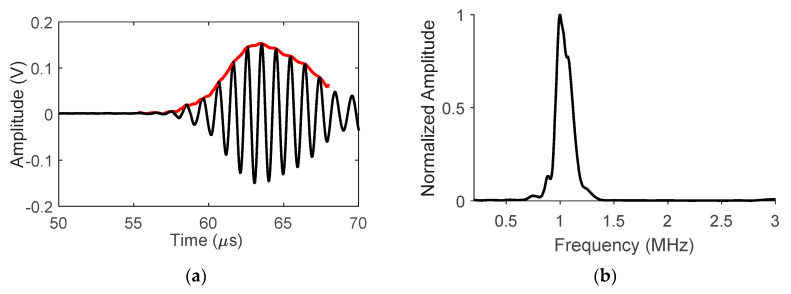

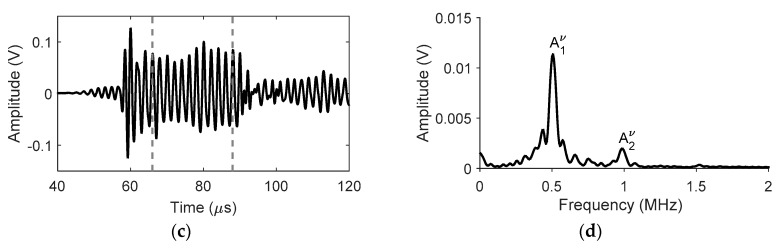
Examples of LUT and NLUT signals (P1) in (**a**,**c**) time domain, and (**b**,**d**) frequency domain.

While the weld heat input has a linear relationship with the weld area, the welding process is complex as there are many other factors that can affect the weld morphology. Unless a visual inspection was conducted, weld heat input did not have any indication of onset of burn-through. Therefore, an additional measurement is needed to understand the initiation of excessive weld penetration, which can be achieved by the UT measurement. In this study, the outputs of LUT and NLUT were UT energy ratio and nonlinearity coefficients, respectively. Examples of LUT and NLUT waveforms (obtained from P1) were shown in Figure 7. The energy ratio of LUT was calculated using the area under the envelope of first arrival waveform, see in Figure 7a [22,23]. The NLUT output, acoustic nonlinearity coefficient ($\beta^{\prime }$), is calculated by the following Equations:

(1) $$\beta =\frac{8A_{2}}{k^{2}x{\left(A_{1}\right)}^{2}}.$$

(2) $$\beta^{\prime }=\frac{A_{2}^{\nu }}{{\left(A_{1}^{\nu }\right)}^{2}}.$$

where $A_{1}$ and $A_{2}$ are amplitudes of fundamental and second harmonics, respectively; $A_{1}^{\nu }$ and $A_{2}^{\nu }$ are voltage amplitudes of the fundamental and second harmonics, respectively; x is the wave propagation distance, which is 10.25 cm; and k is wavenumber. In this case, x and k are the same for all the samples. $\beta^{\prime }$ is rewritten as Equation (2). Figure 7c shows a typical NLUT signal. In order to obtain the frequency spectrum, the proper time window is defined to include only the harmonic signal. The corresponding frequency spectrum is shown in Figure 7d. The fundamental and second harmonic amplitudes are labeled.

**Figure 8:**
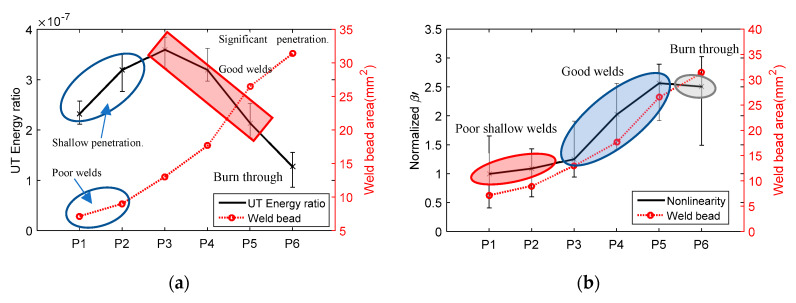
The correlation of weld bead area with (**a**) LUT energy ratio and (**b**) normalized nonlinearity coefficient.

Figure 8a shows the correlation of UT energy ratio and weld bead area. The energy ratio shows an increase from P1 to P3. P1 and P2 have shallow penetration, which can be considered as poor welds. The increase in the amount of solidified material (filler wire and base metal) was considered as the reason for the decrease in UT energy ratio for P3 to P6, which was validated with numerical results presented below. Figure 8b shows the correlation of NLUT nonlinearity coefficient $\beta^{\prime }$ and weld bead area. Except P6, $\beta^{\prime }$ increased with the increase of weld area. P6 had burn-through, which may cause the decay in $\beta^{\prime }$. The NLUT output has more fluctuation (higher error bars) than the LUT output, as the amplitude of second harmonic frequency is influenced more from coupling due to smaller wavelength.

### 3.3. Numerical Results to Support LUT Data

In general, the LUT signal was influenced by (i) the presence of cracks or voids, (ii) scatter at the boundary of base metal and weld metal due to acoustic impedance mismatch and (iii) geometric configuration. Based on the metallographic analysis of the weld samples, no obvious crack or void was observed. Therefore, the numerical models were performed to understand the influences of factors (ii) and (iii) to the LUT signal.

**Figure 9:**
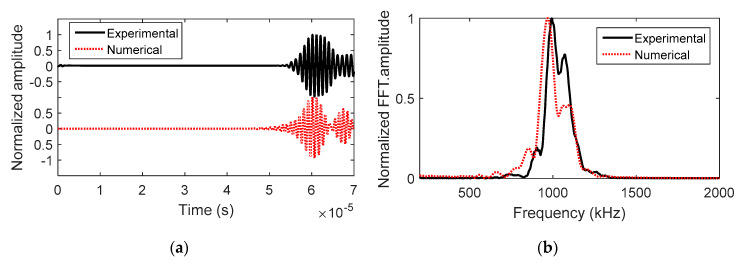
The comparison of experimental and numerical results considering P4 cross section (**a**) time history signals, and (**b**) their frequency spectra.

**Figure 10:**
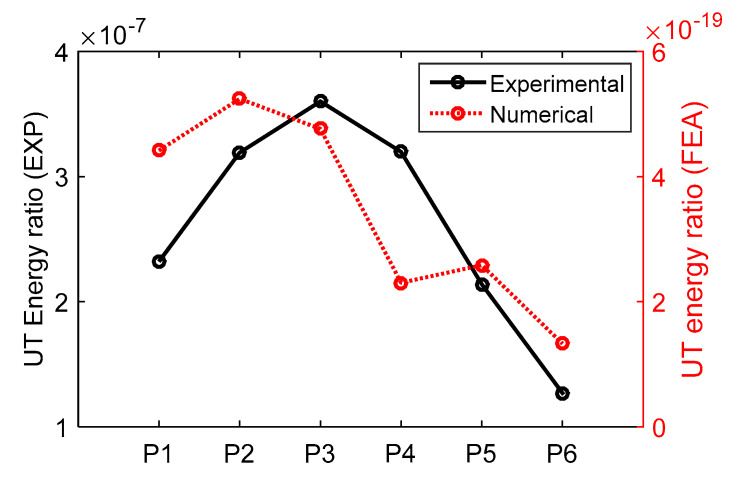
The comparison of numerical and experimental UT energy ratios.

Example waveforms obtained from the experimental and numerical results for P4 were shown in Figure 9. Though differences existed, overall signal shapes in waveforms and their frequency spectra agreed with each other. The main differences can be attributed to numerical simplifications, such as, (a) transducers and couplant were not taken into account; (b) plane strain approximation was used; and (c) materials properties reported in literature were used. In this study, the objective of numerical models was to identify the influences of materials properties and geometric configurations of weld bead to the LUT signal. The LUT energy ratio was extracted from each numerical model, and compared with the experimental measurement (see Figure 10). Similar to the experimental results, the increase of weld bead area correlated with the decrease in the UT energy ratio.

**Figure 11:**
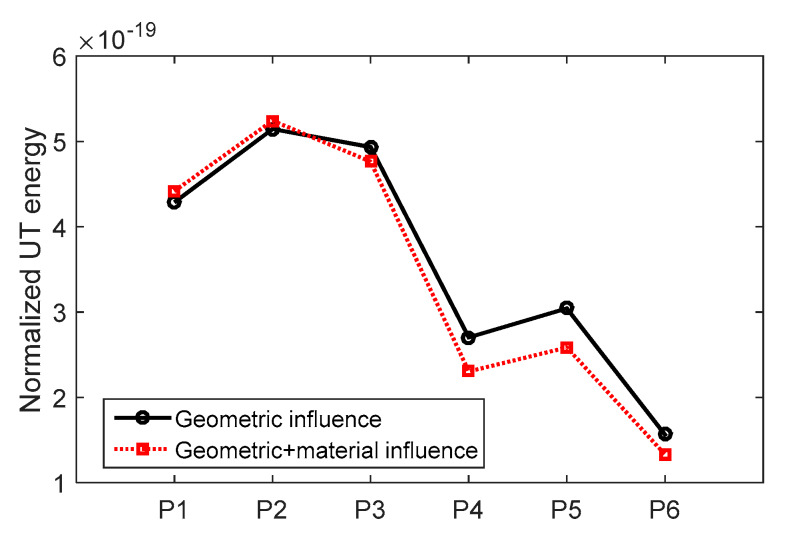
The comparison of geometric influence only and geometric + material influence.

To understand that the decrease in the UT energy ratio was mostly due to geometric configuration rather than materials, the numerical models were repeated such that the same materials properties were used for both base and weld metals. In Figure 11, weld geometry was kept the same for black and red lines while materials properties of base and weld metals were changed. As the acoustic impedances of base and weld metals were close, their interface did not influence the UT output, especially at the selected frequency (1 MHZ used in this study). The influence of weld materials properties on the UT energy ratio was insignificant. The change in the UT energy ratio was mostly attributed to Δh (shown in Figure 5g).

**Figure 12:**
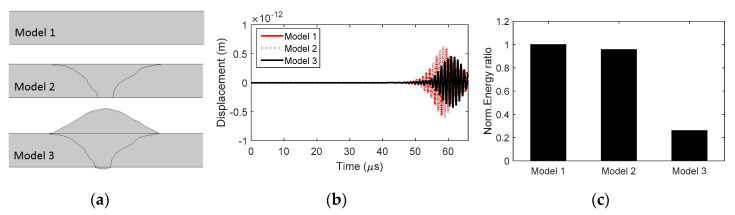
(**a**) The numerical profiles of three models, (**b**) their waveforms and (**c**) the normalized UT energy ratios.

To further understand the influence of weld geometry, three conditions of P5 were modeled: (i) Model 1: Only base metal; (ii) model 2: Weld metal with the same thickness as base metal; (iii) model 3: Entire weld metal and base metal. Three models are shown in Figure 12a. The material properties of base and weld metals defined in Table 3 were considered. The UT signals of models 1 and 2 are similar (Figure 12b) with similar UT energy ratios. However, bead reinforcement at the top and bottom surfaces significantly influences the UT signal and energy ratio: From model 1 to model 2, 4.75% of UT energy ratio decreases; while from model 1 to model 3, 73.9% of UT energy decreases.

**Figure 13:**
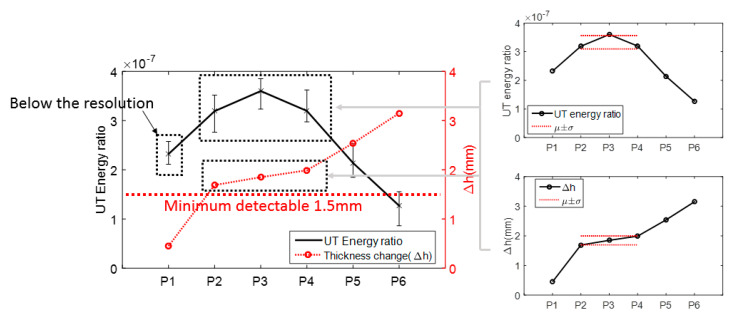
The correlation of thickness change and UT energy ratio.

Based on numerical results, the relationship between UT energy ratio and thickness change (Δh) was established as shown in Figure 13. Considering the detectable size of LUT as one-half wavelength, the minimum detectable size is 1.5 mm for 1 MHz frequency. Usually, the outlier based on the mean ± standard deviation can be assumed as detectable change. The measured UT energy ratios and thickness changes of P2 to P4 were within the boundary, as shown Figure 13. Therefore, the variations of thickness changes of P2 to P4 were difficult to separate. The thickness changes of P5 and P6 were above the resolution of UT measurement.

## 4. Microhardness Results

**Figure 14:**
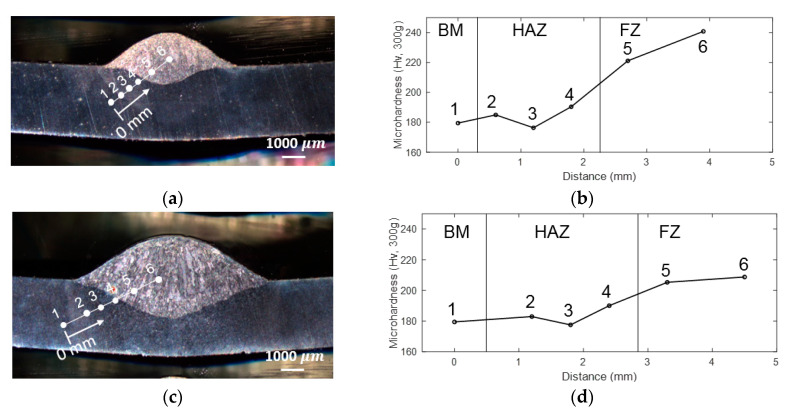

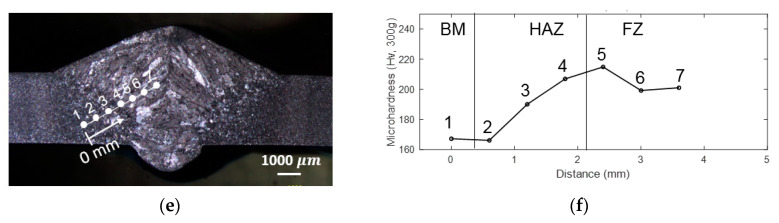
The testing positions of microhardness in (**a**) P1, (**c**) P4 and (**e**) P6 and their microhardness values of (**b**) P1, (**d**) P4 and (**f**) P6. (BM: Base Metal; HAZ: Heat Affected Zone; FZ: Fusion Zone).

In this study, the microhardness measurements were conducted across base metal (BM), HAZ and fusion zone (FZ) for P1, P4 and P6. The measurement points are shown in Figure 14a,c,e. In addition, the corresponding Vickers’s hardness is shown in Figure 14b,d,f. The Vickers measurements show hardness values in the FZ higher than those in the HAZ near the FZ, contrary to what is expected of a low carbon steel weld. This inconsistency is most likely caused by the low amount of base metal molten during welding, due to low heat input, resulting in the molten filler wire being rapidly cooled by the room temperature base metal. Especially for P1 and P4, the metal melting is less due to the low heat input. Whereas the hardness value in FZ is 14–25% higher than the value in BM. This explains that the materials’ properties of base metal and weld metal are close (see in Table 3); meanwhile, the boundary between HAZ and FZ, due to the small thickness, is inconspicuous; therefore, their boundary does not affect the LUT measurement. Note that HAZ is not modeling numerically. In addition, the weld introduced heterogeneity within the weld bead, which can be observed in terms of hardness value differences. By comparing hardness values of P1, P4 and P6 side-by-side, the hardness within the weld bead does not show clear correlation with the weld heat input. Due to sensitivity of NLUT to sub-wavelength defects, NLUT results would be influenced by the heterogeneity in the weld bead as well as the geometric factor.

## 5. Conclusions

This paper aimed to build a quantitative relationship between ultrasonic outputs and weld shape manufactured by GMAW using experimental and numerical models. Macrographs were digitized and inputted into numerical models to capture the weld details in models. The minimum detectable thickness change was controlled by one-half the wavelength of ultrasonic signal. When thickness increases due to weld reinforcement at top and bottom surfaces, ultrasonic energy decreases. The experimental results were validated with numerical models that show that linear ultrasonic output is affected by geometric change rather than materials properties of base metal and weld metal. On the other hand, literature shows that nonlinear ultrasonics is influenced by heterogeneities in medium in addition to geometric pattern. Microhardness results indicate differences in mechanical properties at the heat-affected zone, as compared to base metal and weld metal. Linear and nonlinear ultrasonics can be applied to detect the geometric pattern and the uniformity of weld. In this study, contact transducers were applied. Non-contact ultrasonic transducers can be adapted at lower frequency (~500 kHz) with the reduced resolution.

In summary, the main conclusions of this paper can be grouped into three aspects:

(1) The numerical models connected with macrographs through digitized images provide an accurate representation of weld morphology. The numerical results show that LUT is influenced by the presence of weld exceeding the base plate thickness, other than the materials’ properties of weld, as compared to base metal.
(2) The experimental and numerical results show that excessive weld penetration causes a decrease in the ultrasonic energy. Heat input increases with weld penetration. However, unless a visual inspection is conducted, or it is known that heat input is excessive, the excessive weld penetration cannot be predicted by only heat input. UT provides a quantitative and automated assessment of weld quality.
(3) While LUT is influenced only by geometric variability of weld morphology, NLUT shows the dependence on both geometry and heterogeneity. The two methods can be used concurrently for more accurate assessment of weld quality.

## Figures and Tables

**Table 1:** Examples of Ultrasonic Testing (UT) studies for weld inspection.

Application	Experimental Configuration	Material	Weld	Reference
Evaluate and visualize V-butt joint weld	Ultrasonic-based tomography	Austenitic material	V-butt joint	[8,9]
Detect the internal defects hidden inside butt-joint weld	Linear phase array ultrasonic technique	Chromium-zirconium copper	Butt-joint weld	[10]
Identify the simulated weld defects by Lamb wave	Linear ultrasonic transducer with variable angle probes (through-transmission)	Steel	Simulated weld (no actual weld)	[11]
Assess the microstructure change	Immersion UT	Duplex stainless steel	GMAW (Gas metal arc welding)	[12]
Correlate UT measurement to the mechanical properties within the weld bead	Immersion UT	Stainless steel 304L	V-butt joint	[13]
Evaluate microstructural deterioration due to thermal aging treatment	Pulse-echo UT measurement	625 nickel-based alloy	Butt weld	[14]
Evaluate the weld quality in-situ	Embedded the ultrasonic probe into the electrode in pulse-echo mode	Dual-phase steel DP590	Resistance spot weld	[15]
Identify the shrink-cased defect in the weld bead	Multi-transducer guided wave in pulse-echo mode	Stainless steel 316	Weld joint	[16]
Identify the kissing bond flaws in welded butt joint	Nonlinear ultrasonic method in through-transmission mode	Aluminum alloy	Butt-joint weld (friction stir welding)	[17]
Identify the range of HAZ (Heat affected zone)	Nonlinear ultrasonic method in through-transmission mode	Stainless steel 304	V-butt joint	[18]
Test the mechanical performance of weld joint	Linear ultrasonic by Lamb wave	Aluminum alloy	Weld joint	[19]

**Table 2:** The specifications of weld samples.

Sample	Wire Speed (mm/s)	Gas Flow Rate (Liter/min)	Travel Speed (mm/s)	Heat Input (kJ/mm)
P1	42.3	18.83	11	0.18
P2	50.8	18.83	11	0.20
P3	67.7	18.83	11	0.30
P4	84.7	18.83	11	0.38
P5	101.6	18.83	11	0.49
P6	118.5	18.83	11	0.58

**Table 3:** Materials’ properties (based on ASTM A1008/A1008M [21]) used in the numerical models.

Property	Base Metal	Angled Wedge (Acrylic Plastic)	Weld Metal
Young’s Modulus (GPa)	190	50.8	205
Poisson’s ratio	0.28	0.34	0.295
Density (kg/m^3^)	7800	1160	7850

**Table 4:** The summary of weld dimensions as a result of different weld heat inputs.

Sample	Width (mm)	Bead Height (BH) (mm)	Bead Penetration (BP) (mm)	Area of Weld Bead (mm^2^)	Weld Heat Input (kJ/mm)
P1	5.96	0.45	0.83	7.14	0.18
P2	6.54	1.69	0.89	9.01	0.20
P3	7.70	1.85	1.41	13.01	0.30
P4	8.67	1.99	1.66	17.65	0.38
P5	9.27	2.18	3.56 *	26.54	0.49
P6	9.84	2.13	4.22 *	31.38	0.58

* penetration through the thickness.
